# Optimization and Validation of HS-GC/MS Method for the Controlled Release Study of Microencapsulated Specific Bioattractants for Target-Plaguicide Production

**DOI:** 10.3390/molecules26040996

**Published:** 2021-02-13

**Authors:** María Luz Alonso, Oskar González, Rosa María Alonso

**Affiliations:** Analytical Chemistry Department, Faculty of Science and Technology, University of the Basque Country (UPV/EHU), Barrio Sarriena s/n, 48940 Leioa, Spain; oskar.gonzalezm@ehu.eus

**Keywords:** microencapsulated products, volatile compounds, DSC, HS-GC/MS, controlled release

## Abstract

Insect plagues are a problem often hard to solve due to the harmful effects caused by the pesticides used to combat them. Consequently, the pesticide market is increasingly trying to develop new technologies to prevent the unwanted effects that common plague treatments usually bring with them. In this work, four specific bioattractants of *Musca domestica*, extracted from fungi (β-ocimene, phenol, p-cresol, and indole) were microencapsulated with β-cyclodextrin in order to produce an economically and environmentally sustainable bait containing biocides in the near future. Cyclodextrins will retain these volatile compounds until their use by the consumer when the product comes into contact with water. Then, the bioattractants will be released in the medium in a controlled manner. An analytical methodology based on headspace extraction coupled to gas chromatography and mass spectrometry (HS-GC/MS) has been developed and validated following Environmental Protection Agency (EPA) and European Commission Directorate General for Health and Food Safety guidelines for the bioattractants controlled release study from the microencapsulated product. The analytical method has been shown to be accurate and precise and has the sensitivity required for controlled release studies of the four bioattractants analyzed. The release of the bioattractants from microencapsulated products achieved the “plateau” after 3 h in all cases.

## 1. Introduction

Environmental health focuses its attention on the relationships between humans and their environment and tries to improve human health and well-being. This science has as objective the establishment of policies and programs to reduce chemical and other environmental exposures in air, water, soil, and food to protect people and provide communities with healthier environments [1]. One of the great challenges of the 21st century is the search for alternative methods associated with the use of chemical substances, which not only preserve the natural equilibrium but also aim to decrease their toxicity level. Among the environmental health problems, arthropods and especially *Musca domestica* are found. The fly breeding consists of animal wastes and other organic compounds, being important carriers of disease-causing microorganisms, such as salmonella, typhoid, and other diarrheal diseases [2]. In addition, these insects have developed a resistance to certain pesticides [3,4].

**Figure 1:**
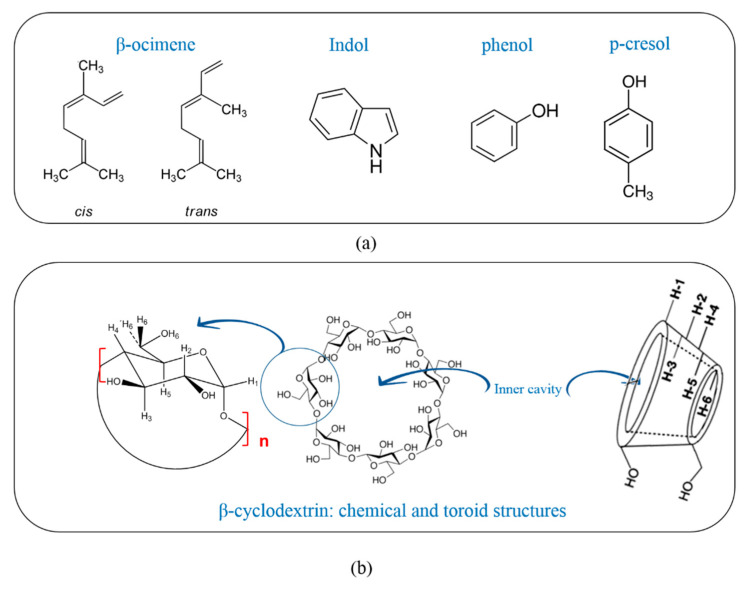
(**a**) Guest molecules structure: cis and trans β-ocimene, indole, phenol and p-cresol. (**b**) CD chemical and toroid structures, *n* = 7.

In order to find a possible solution to these problems, different strategies have been proposed. It is known that some fungi and plants are able to release volatile organic compounds (VOCs) to the environment. In fact, concerning the way we use them, VOCs can act not only as pesticides, but many of them can also play important roles as signaling molecules between species [5,6,7]. As an example, there are plants and fungi in nature that spew volatile chemicals that attract flies by smell [8]. In the literature, works have been found where it is reported that some natural essential oils produce odors that attract flies in search of a food source or medium in which to deposit their eggs. Some of these volatile attractants can also operate as pesticides, such as indole, which works as an attractant and as a repellent depending on its concentration [9,10]. Moreover, there are chemical attractants that are available on the market with zero lethality and can be used as insect attractants, i.e., pheromones as (*Z*)-9-tricosene or fatty acids [3,5,6,7,8,9,10]. According to this information, the extraction and analysis of some VOCs released by fungi, their function known as *Musca domestica* bioattractants, were carried out in our laboratory. As a result of this work, VOCs, β-ocimene, indole, phenol, and p-cresol (Figure 1a) were identified and the attraction assays done using *Musca domestica* showed their bioattractant function. Considering this, it would be expected that the combination of this type of attractants with biocides could generate more effective and selective products against the target organism [11,12,13,14,15,16,17,18].

Nowadays, in the biocides field, there is a great concern on the reduction of the most extended used pesticides because of their high toxicity [19], due to the extremely harmful effects caused to both, environment and human health. Their use is strictly controlled by international organizations, such as EPA (Environmental Protection Agency 1996), World Health Organization (WHO 1996; 2006), Food and Agriculture Organization of the United Nations (FAO 2018) and the European Commission Regulation (EC 2005; 2009), limiting not only their use but also the Maximum Residue Levels (MRL) on animal feed and human food [20,21,22,23,24,25]. However, the fact is that, apart from the strict control of the use of these toxic substances, in the last decades, there is an increasing trend to develop new technologies in order to produce more sustainable products to substitute the previous ones. At this point, the use of volatile bioattractants for the target insect in the final product would reduce the use of pesticides. In order to preserve these volatile compounds until their use by the consumer and to control their release in the medium, the microencapsulation technique has proved to be a good alternative.

The microencapsulation process is carried out using different types of encapsulating agents, such as inorganic materials, lipids, proteins, gums, polymers, and carbohydrates [26]. Among them, the cheapest carbohydrate ß-cyclodextrin (CD), Figure 1b, is an encapsulating agent widely used to produce volatile controlled release, therefore, it was chosen for the microencapsulation of the bioattractants studied in this work. The hydrophilic inner cavity of cyclodextrins can host hydrophobic molecules to form host-guest complexes in which the guest molecule is encapsulated by the CD forming the called inclusion complexes, either in solid phase or in aqueous solution [26,27,28,29,30,31,32,33,34,35]. 

The research undertaken in this work is focused on synthesizing an efficient, specific, and sustainable bait against *Musca domestica* by means of a microencapsulation process, using bioattractants and biodegradable cyclodextrins as encapsulating agents. This technology allows the use of a small amount of the active compounds and their controlled release. Moreover, it provides greater stability and decreases the volatility and toxicity of the microencapsulated compounds [36]. The substances entrapped within the microcapsules are protected from oxidation and degradation, oxygen-sensitive and photosensitive substances are stabilized, and unwanted odor can be masked. Besides, it is possible to diminish the concentration of the active substances released to the environment, resulting in reduced toxicity [37,38]. Furthermore, this emerging technology makes it possible to produce environmentally friendly as well as economical products, which are not only sustainable but also socially accepted.

The slower the release, the lower will be the amount of active ingredients available for leaching and volatilization. This phenomenon depends on kinetic and thermodynamic parameters. As interactions between phases occur, distribution and diffusion coefficients of the encapsulated compound must be taken into account since they will largely determine the release profile of the compound from the inside, across the CD matrix, until they get outside [26,39,40]. For phenol family of bioatractants, Van der Waals forces [27] and hydrogen bonds must also be considered in this phenomenon.

The release study of the microencapsulated product synthesized will be carried out in water, which would be added right at the time of the use of the bait, when the process of attractant releasing begins [41,42]. The kinetics of release from bioattractants: CD inclusion complex was determined from the best fit of the release data to Fickian and non-Fickian diffusion using Higuchi and Ritger–Peppas models [43,44,45].

In previous works of the research group, biocides used against the plague of *Musca domestica* were microencapsulated [1,18] and bioattractans as β-ocimene, phenol, p-cresol, and indole were extracted from fungi [17]. In the future, it will be possible to synthesize an efficient, specific, and sustainable bait against *Musca domestica* containing both biocides and bioattractants. For this purpose, it will be necessary to study the potentiality of inclusion complex formation with cyclodextrins to retain *Musca domestica* bioattractants. Therefore, the aim of this work is to characterize the microencapsulation product and optimize and validate an analytical methodology for the determination of bioattractants in the release study. The chosen technique for characterization of the microencapsulated product was differential scanning calorimetry (DSC), and headspace gas chromatography coupled to mass spectrometry (HS-GC/MS) was used for the release study. 

## 2. Results and Discussion 

### 2.1. Microencapsulation Characterization.by Differential Scanning Calotimetry (DSC) 

**Figure 2:**
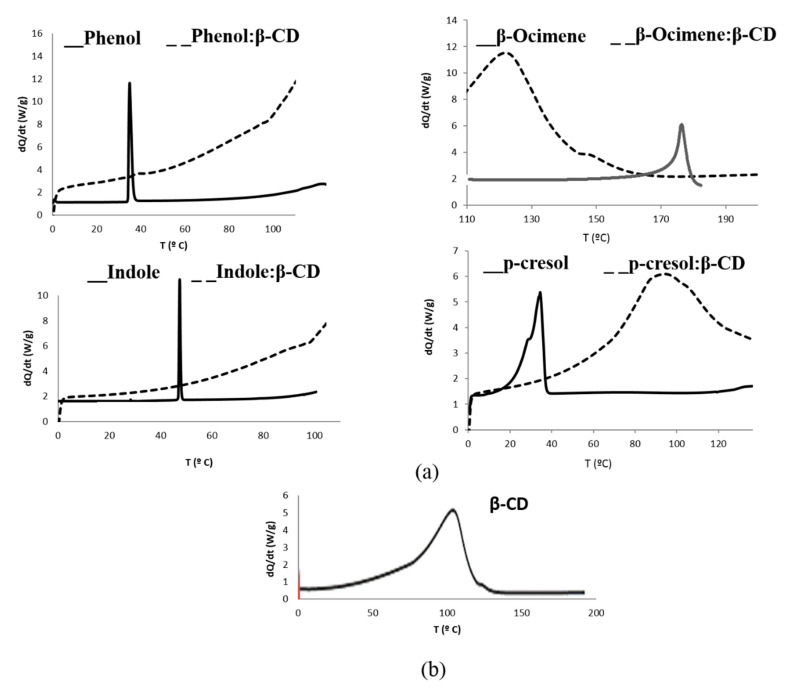
(**a**) Thermograms obtained for free (__) and microencapsulated biottractans (- - -) and (**b**) β-CD.

Encapsulation involves the disappearance of the characteristic peak of each bioattractant in the inclusion complex thermogram. This peak usually corresponds to the melting point of the substance analyzed, except for β-ocimene, which is liquid and in that case, the characteristic peak followed is its boiling point. Peaks at 175.2, 40.9, 35.5, and 52 °C for β-ocimene, phenol, p-cresol, and indole, respectively, were obtained (Figure 2a). Moreover, an endodermic peak at 111.2 °C for CD associated with loss of humidity could be observed (Figure 2b). Inclusion complex thermograms demonstrate that, in all cases except for phenol, no biottractant characteristic peak was observed, thus, the bioattractants were within cyclodextrin matrix. Phenol is also encapsulated, although a small peak appears at 40.9 °C, probably due to the existence of a small concentration of phenol in the air [46,47].

### 2.2. Optimization and Validation of HS-GC/MS Method for Bioattractants Analysis in the Microencapsulated Product

**Figure 3:**
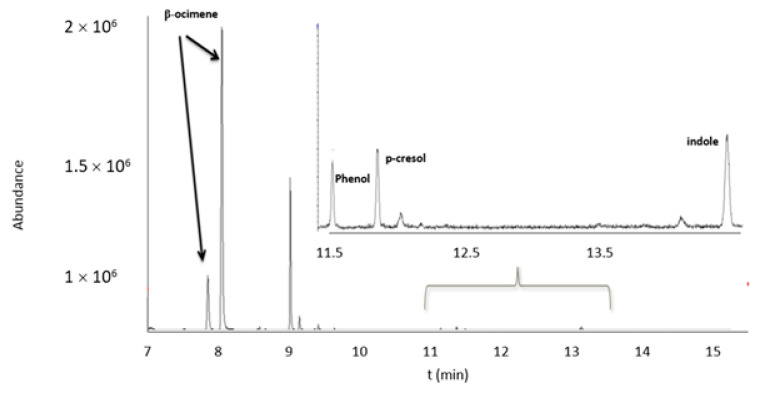
Chromatogram obtained from analysis by HS-GC/MS of the mixture of bioattractants (β-ocimene, phenol, p-cresol, and indole) in concentration 10 mg/L.

In Figure 3, a chromatogram obtained for a bioattractant multicomponent solution of 10 mg/L is shown. A good chromatographic separation was obtained in the optimized chromatographic and mass detector conditions, taking into account the characteristic ions of each compound (Table 1).

**Figure 4:**
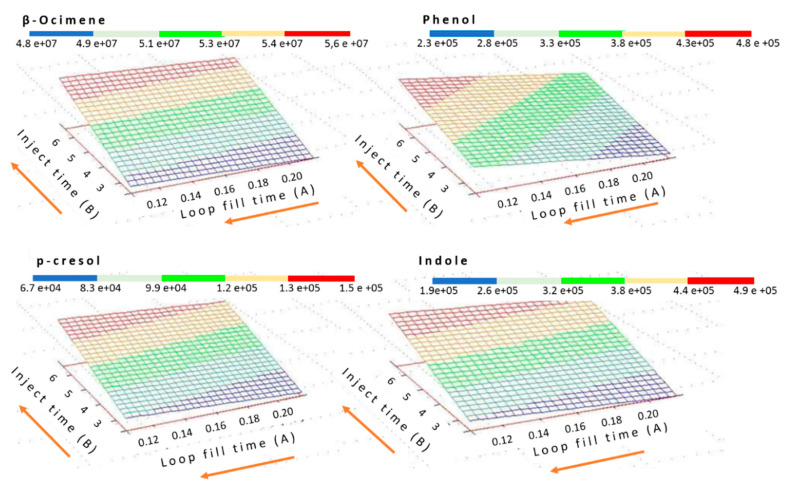
Response surfaces plots using central composite design (CCD), obtained for each bioattractant for injection time and loop fill time optimization of HS-GC/MS.

The optimization of injection time and loop fill-time parameters of the HS-GC/MS method was performed by using central composite design (CCD). Nine experiments and two replicates of the same point were executed [48] (Table 2). Figure 4 shows the response surfaces obtained for each compound for injection time and loop fill time optimization. 

**Figure 5:**
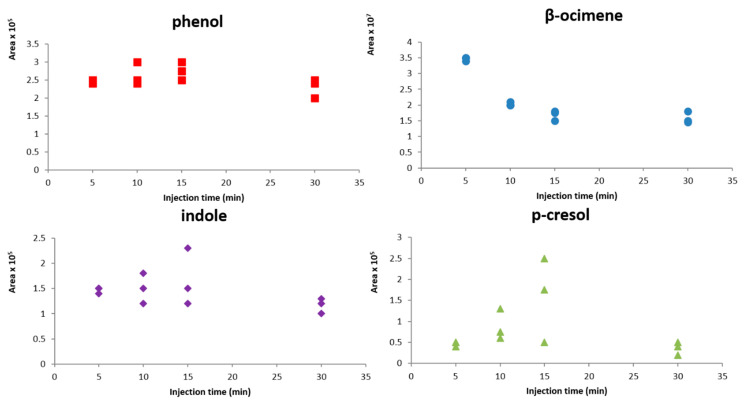
Variation of chromatographic peak area of each bioattractant with injection time (three replicates).

From the results obtained from response surfaces, the injection time was set at 6 min and the loop fill time was 0.10 min. Despite these results, it is observed that the trend of the response surface for all bioattractans was increasing. Therefore, it was decided to extend the study of the injection time effect on the response already obtained, using higher injection times. Time values of 10, 15, and 30 min were also tested. As can be seen in Figure 5, the increase of injection time does not give rise to a significant increment of sensitivity for all the bioattractants, therefore, an injection time of 6 min was chosen as optimum due to the repeatability obtained. 

**Figure 6:**
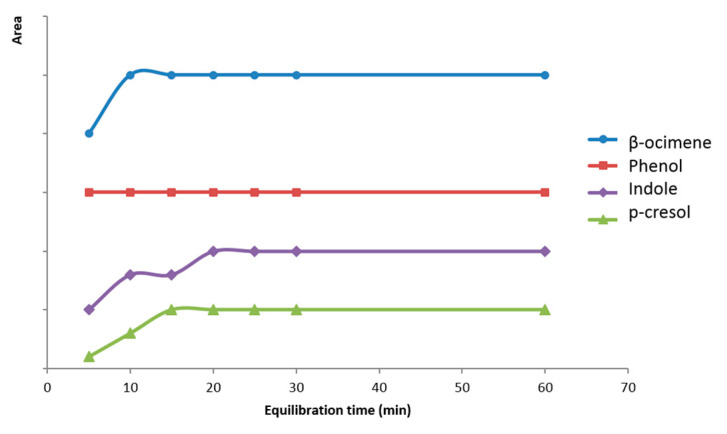
Variation of chromatographic peak area with equilibration time for each volatile compound.

Once the optimum injection time was established, the equilibration time assay was performed. Times ranging from 5 to 60 min were tested. As can be seen in Figure 6, from 20 min of equilibration time chromatographic peak areas became practically constant for all compounds. However, 20 min of equilibration time could be insufficient for bioattractants to go out from the cyclodextrin inclusion complex and equilibrium between liquid and gaseous phase might occur, therefore, in order to assure the process, time was extended to 60 min.

**Figure 7:**
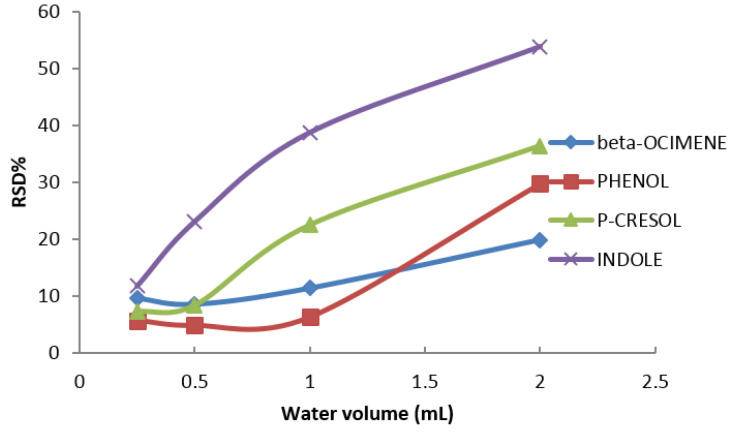
Influence of water volume added to encapsulated product on the repeatability of the method in terms of RSD%.

Finally, taking into account the bibliography, the octanol/water-partitioning coefficient (k_ow_) for monoterpenes like β-ocimene can vary Henry’s law constant value, and thus, alter the mass transference of the compound between liquid and gas phase [49]. Therefore, the effect of water volume on the repeatability of the method was studied. As can be seen in Figure 7, the lowest water volume (0.25 mL) offers the best repeatability (RSD% < 15), hence, this water volume was chosen throughout the research.

**Figure 8:**
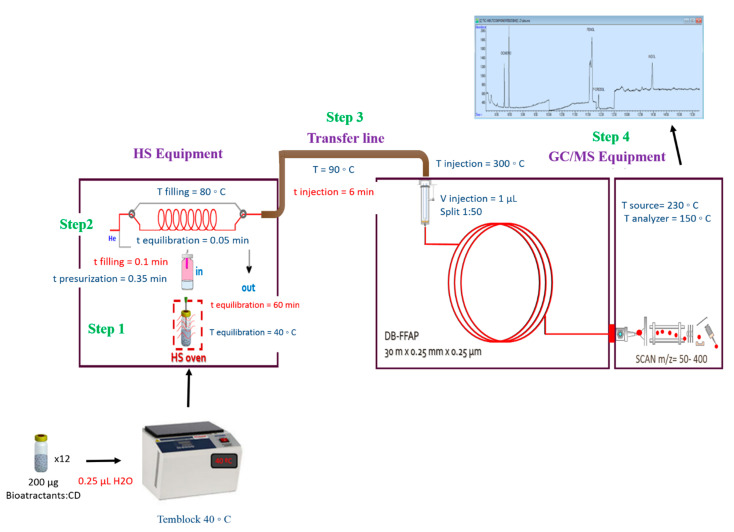
Scheme of the HS-GC/MS methodology used for the study of the controlled release of the microencapsulated bioattractants. Step 1: liquid-gas equilibrium stage. Step 2: gas equilibration time, Step 3: gas goes ahead the transfer line to the gas chromatograph, Step 4: separation of the analytes from the sample on chromatographic column and quantification of the analytes.

In Figure 8, a scheme of HS-GC/MS system used for monitorization of bioattractants including the optimal conditions is shown.

The optimal HS-GC/MS followed consisted on: firstly 200 µg of each microencapsulated bioattractant were set in 12 closed vials and 0.25 mL of water was added. All the samples were prepared at the same time. One of the vials was measured at the initial time. The rest of the vials were placed in a termblock at 40 °C and they were analyzed at different times to determine the release profile, every hour up to seven hours and then, once a day until a week. In step 1, the vial with the sample is introduced into the equipment and kept at 40 °C for 60 min. This is the liquid-gas equilibrium stage. Once elapsed, step 2 occurs, where the vial is pressurized for 0.35 min before filling the loop for 0.05 min at 80 °C. After an equilibration time of 0.1 min, it goes ahead the transfer line to the gas chromatograph for 6 min at 90 °C in step 3. Finally, in step 4 the separation of the sample analytes on the indicated chromatographic column and the quantification of bioattractants take place. Injection port used was 300 °C and only 1 uL of sample was injected with a 1:50 flow division. The ionization source and the quadrupole used were 230 °C and 150 °C, respectively. Mass scan was 50–400 m/z.

Once the HS-GC/MS was optimized, the validation of the method was accomplished by following the SANTE/12682/2019 guidance document. LOQ was 0.1 mg/L for indole, 0.15 mg/L for phenol and p-cresol, and 0.2 mg/L for β-ocimene. The linear concentration range obtained for the four volatile compounds was from LOQ to 1 mg/L. In terms of lineality, Pearson’s correlation coefficient was 0.995 for ocimene, 0.997 for phenol, and 0.999 for indole and p-cresol. The distribution of the residuals did not follow any trend and the relative error of all the calibration standards was lower than 20%. As can be observed in the recovery and RSD values shown in Table 3, the method fulfilled the intra- and inter-day validation criteria for trueness (70–120% recovery) and precision (<20% RSD).

### 2.3. Microencapsulated Bioattractants Controlled Release Study 

**Figure 9:**
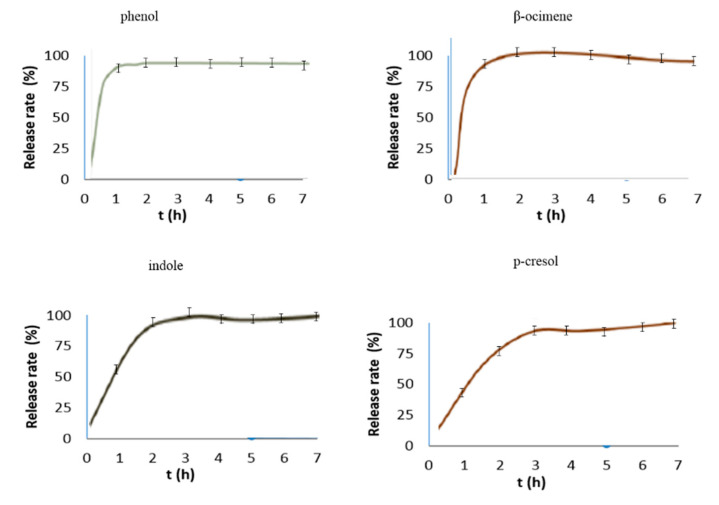
Controlled release rates of the bioattractants from microencapsulated product.

The release profile obtained for the microencapsulated bioattractants can be observed in Figure 9, as well as the average profile of three replicates for each bioattractant. The release rate was carried out following the validated method shown in Figure 9. Only the first seven hours are represented because it remains constant until one week. 

Profiles obtained revealed that each bioattractant showed different release rates over time. After three hours, a maximum release has been reached. After that time, a plateau was achieved. In view of these results, a sustained release is achieved over time, which promises good attraction results. 

The release study data were analyzed on the basis of Fickian and non-Fickian release kinetics. The release rates k and n of each model were calculated by linear regression analysis. Coefficients of correlation (r^2^) were used to evaluate the fit of the data. A plot of β-ocimene, phenol, p-cresol, and indole for Higuchi equation [45] resulted in straight line with r^2^ values of 0.9865, 0.9854, 0.9992, and 0.9858, respectively and k value were 0.51, 0.50, 0.50, and 0.54, respectively. The values calculated suggested that the release of the bioattractant from the inclusion complex occurred as a square root of time-dependent process based on Fickian diffusion. 

## 3. Material and Methods

### 3.1. Material

CD (Cavamax®W7) was acquired from Wacker Fine Chemicals (Munich, Germany) with a purity higher than 95%. The bioattractants β-ocimene (*E* and *Z* isomer mixture, >90%), phenol (>99%), p-cresol (99%), and indole (99%) of analytical reagent quality were delivered by Sigma-Aldrich® (St. Louis, MO, USA). Methanol and acetone used were HPLC grade (>99.9%) and purchased to Teknokroma® (Barcelona, Spain). Ultrapure water (18.2 MΩ/cm, 25 °C) was obtained from Milli-Q® Element A10 system (Millipore, Milford, MA, USA). 

Solutions of the four bioattractants at a concentration of 10 and 1 mg/L in acetone were prepared for HS-GC/MS method validation. For the study of linearity, calibration curves were constructed in the range between 0.1 and 1 mg/L with the corresponding blank.

A Sartorius analytical balance CP224S from Sartorius Mechatronics (Goettingen-Germany) was used for weighing the solid reagents. Freeze-drying equipment (FreeZone Plus 12 Liter, Labconco, KS, USA) was employed to evaporate water from the microencapsulated product. DSC thermograms were obtained by using a Mettler Toledo DSC 822® calorimeter (Barcelona, Spain) calibrated with indium. The chromatographic analysis was performed using an Agilent 7694E headspace autosampler coupled to Agilent 7820 gas chromatograph and a 5975C mass spectrometric detector (Agilent Technologies, Palo Alto, CA, USA). A DB-FFAP (30 m × 0.25 mm × 0.25 μm) chromatographic column was used. Helium was the carrier gas. Source and MS analyzer temperatures were fixed at 230 °C and 150 °C, respectively. A Tembloc system (Selecta, Barcelona, Spain) was also used to keep the samples at 40 °C to controlled release studies.

### 3.2. Methods

#### 3.2.1. Microencapsulation Procedure 

The microencapsulation procedure used was the one optimized previously by our research group [16,18]. The bioattractant dissolved in acetone was added to an aqueous solution of β-cyclodextrin in a molar ratio of 1:1 and shaken for 15 min. Then, it was evaporated in freeze-drying equipment. Physical mixtures of each bioattractant with CD were prepared by grinding equal amounts of both compounds in a mortar.

#### 3.2.2. Microencapsulation Characterization.by Differential Scanning Calorimetry (DSC)

DSC thermograms of pure bioattractants, pure cyclodextrin, physical mixture of each bioattractant with CD and the encapsulated compounds were recorded to confirm that the encapsulated process had taken place. All the analysis were done under the same experimental conditions using the STARe program system. The temperature scale was calibrated using indium as reference. Samples of around 10 mg were put into aluminum pans and covered with lids, which were pierced to permit the gas release during the heating process. DSC scans were performed in duplicate, under dynamic nitrogen atmosphere (50 mL/min) and at a heating rate of 10 °C/min (0–200 °C) under nitrogen atmosphere (80 cm^3^/min).

The disappearance of bioattractant peak in DSC thermograms at its characteristic melting point (40.9 °C, 35.5 °C, and 52.0 °C, for phenol, p-cresol, and indole, respectively) or the boiling point (175.2 °C for β-ocimene) will be indicative the microencapsulation process has taken place. 

#### 3.2.3. Optimization and Validation of HS-GC/MS Method for Bioattractants Analysis in the Microencapsulated Product

This study was focused on the measurement of the free volatile compounds from encapsulated compounds with CD. Solutions of the four bioattractants at a concentration of 1 mg/L were added to different 10 mL vials containing 2 mL of water and CD in molar ratio 1:1. Blank, only with CD in water, was also performed. 1 uL volume was injected in 1:50 split mode at 1.2 mL/min constant flow in the HS-GC/MS system. The temperature program was optimized at an initial temperature of 50 °C for 4 min, followed by a ramp to 45 °C/min to 220 °C, holding for 5 min. The injector temperature was set to 300 °C, while the temperature of the source detector was 230 °C.

SCAN mode, for solutions of 10 mg/L of each compound separately, was chosen to identify the characteristic ions (m/z) of the compounds studied. Secondly, a multicomponent solution of 1 mg/L was introduced in SIM mode, measuring the most abundant ions (*m/z)* in the mass spectrum.

In the HS-GC/MS system, the following parameters were optimized: Injection time, the loop fill time, and equilibration time. Oven, loop, and line transfer temperatures were previously defined, fixing them at 40 °C, 80 °C, and 90 °C, respectively [49,50]. Based on previous work done by the research group, the equilibration time of the vial for solutions of these compounds in methanol was set at 10 min [17]. Therefore, firstly, it was decided to optimize the injection and the loop fill times by a central composite design (CCD), using The Unscrambler® v. 9752 program [48] for the design and treatment of the results. The time intervals were established between 0.10 and 0.22 min for loop fill time and 2.4–6.6 min for injection time, following the equipment instructions manual. Once they were fixed, equilibration time was calculated. This is the most important parameter because it is necessary to establish the optimum time in which the amount of compounds is stable in the headspace. To optimize this parameter, several injections at different equilibration times, from 5 to 60 min every 5 min, were done.

After optimization of the HS-GC/MS system, the analytical method was validated. Validation of the method was implemented by following the criteria of the Environmental Protection Agency [51] and analytical quality control and validation procedures guide for pesticide residue analysis (SANTE/12682/2019) [52].

LOQ was calculated using the equation (yblank + 10 × σblank), where yblank is the average of the areas obtained for the blank solution and σblank the standard deviation of these areas. 

The linear range was established based on previously calculated bioattractant concentrations in fungi by the research group [17]. Furthermore, these volatile compounds are attractive at low concentrations, whereas at high concentrations, they may become repellent [18]. Calibration curves consisted of a blank and a minimum of five calibration standards were constructed in the range between LOQ and 1.0 mg/L by plotting the peak area of the analyte against its nominal concentration. Since the calibration range was relatively narrow, a simple linear regression method was applied. The fit of the calibration function was inspected visually and by calculation of the residuals. The acceptance criterion for the calibration curve was that the calculated concentration of all the calibration standards should be ±20% of the nominal value. 

For the determination of intra-day trueness and precision, six replicates at three different concentration levels were measured. The lowest level corresponds to the LOQ, the medium level is located in the intermediate calibration zone (0.5 mg/L) and the highest level corresponds to the highest concentration (1 mg/L) of calibration curve. Precision is expressed in terms of relative standard deviation (RSD%), while recovery was used as a measure of trueness. Inter-day trueness and precision were also calculated by performing the experiments by triplicate on three different days.

#### 3.2.4. Microencapsulated Bioattractants Controlled Release Study

Microencapsulated samples were prepared separately for each bioattractant for the release study and analyzed by the optimized and validated HS-GC/MS method. First, 200 µg of each microencapsulated bioattractant were set in 12 closed vials. Then, 0.25 mL of water was added. All the samples were prepared at the same time. One of the vials was measured at the initial time. The rest of the vials were placed in a temblock at 40 °C and they were analyzed at different times to determine the release profile, every hour up to seven hours and then, once a day until a week.

After measuring the concentration of the released microencapsulated bioattractants by HS-GC/MS, the release ratio (%) of each one was determined, based on Equation (1), where the time-dependent released amount M_t_ is divided by the total amount of bioattractant M_tot_, initially inside the inclusion complex.

Released compound % = M_t_ × 100/M_tot_ (1)

From the bioattractant amount measured by HS-GC/MS method, the experimental amount of M_t_ was determined. The calculation of the total amount located (guest) in the inclusion complex, (M_tot_) was done by gas chromatography coupled with mass spectrometry (GC/MS). The analysis was performed under the same parameters optimized and validated for HS-GC/MS method. In that case, the samples were prepared with microencapsulated products and methanol as a solvent instead of water. Methanol was used due to the highest solubility of these bioattractants in this solvent.

## 4. Conclusions

In this work, for the first time to our knowledge, β-cyclodextrin has been used as an encapsulating agent of the bioattractants of *Musca domestica.* β-ocimene, phenol, p-cresol, and indole and the microencapsulation process were verified by DSC technique. 

Cyclodextrins will retain these bioattractants until used by the consumer, when the product comes into contact with water. Therefore, in this work, a study of the behavior of the release of these volatile bioattractants from the interior of cyclodextrin was carried out, once water was added. For this purpose, a HS-GC/MS method was developed to monitor the release of each volatile compound at 40 °C.

The HS-GC/MS method was optimized and validated following EPA and SANTE guidelines for the bioattractants controlled release study from a microencapsulated product. The analytical method has been shown to be accurate and precise and has the sensitivity required for controlled release studies of the four bioattractants studied. 

From the results obtained in this research, rapid fabrication of a microencapsulated product with β-cyclodextrin containing *Musca domestica* bioattractants: β-ocimene, phenol, p-cresol, and indole can be proposed. The specific volatile compounds are retained thanks to the cyclodextrin. Only with the addition of water, bioattractants will be released in a controlled mode (3 h), attracting the *Musca domestica*. 

The final microencapsulated product will be manufactured together with pesticides in the future in order to make bait. Therefore, it will be at this time when it will work to control the plague. This bait would be an effective and selective product to the target insect, decreasing the amount of biocide, and therefore, the impact on the environment and human health.

## Figures and Tables

**Table 1:** Experiment matrix for CCD.

Compound	Retention Time (min)	*m/z*
β-ocimene	8.02/8.23	79, 91, 93
Phenol	11.47	39, 66, 94
p-cresol	11.71	107, 108
Indole	13.56	90, 117

**Table 2:** Experiment matrix for CCD.

Experiment	Injection Time (min)	Loop Fill Time (min)
1	4.50	0.10
2	4.50	0.22
3	2.40	0.16
4	6.60	0.16
5	3.00	0.12
6	3.00	0.20
7	6.00	0.12
8	6.00	0.20
9	4.50	0.16
10	4.50	0.16
11	4.50	0.16

**Table 3:** Validation parameters of HS-GC/MS method used for the study of the controlled release of the microencapsulated bioattractants.

Compund	Concentration (mg/L)	Recovery %		RSD %	
		Trueness Inter-Day	Trueness Intra-Day	Repeatability Inter-Day	Repeatability Intra-Day
β-ocimene	0.2 (LOQ) 0.5 1	103.2 116.1 109.9	105.9 115.1 109.1	13.0 9.8 13.5	13.4 7.8 13.0
Phenol	0.15 (LOQ) 0.5 1	98.2 98.4 91.3	98.9 99.1 94.4	8.6 9.1 8.5	3.3 10.2 7.5
p-cresol	0.15 (LOQ) 0.5 1	91.1 94.3 95.6	90.5 92.7 89.3	8.4. 7.9 16.7	2.4 9.0 10.7
Indole	0.1 (LOQ) 0.5 1	106.9 106.4 97.6	107.7 106.8 94.0	9.2 6.5 13.4	9.5 6.2 14.9

## Data Availability

Data which support the results obtained in this study are confidential since a contract has been signed with a chemical company.
